# Evaluating convergence between two data visualization literacy assessments

**DOI:** 10.1186/s41235-025-00622-9

**Published:** 2025-04-05

**Authors:** Erik Brockbank, Arnav Verma, Hannah Lloyd, Holly Huey, Lace Padilla, Judith E. Fan

**Affiliations:** 1 Department of Psychology, Stanford University, Stanford, USA; 2 Department of Psychology, University of California San Diego, La Jolla, USA; 3 Department of Computer Science, Northeastern University, Boston, USA

**Keywords:** Graph comprehension, Graphical literacy, Data literacy, Psychometric evaluation, STEM education

## Abstract

Data visualizations play a crucial role in communicating patterns in quantitative data, making data visualization literacy a key target of STEM education. However, it is currently unclear to what degree different assessments of data visualization literacy measure the same underlying constructs. Here, we administered two widely used graph comprehension assessments (Galesic and Garcia-Retamero in Med Dec Mak 31:444–457, 2011; Lee et al. in IEEE Trans Vis Comput Graph 235:51–560, 2016) to both a university-based convenience sample and a demographically representative sample of adult participants in the USA (*N*=1,113). Our analysis of individual variability in test performance suggests that overall scores are correlated between assessments and associated with the amount of prior coursework in mathematics. However, further exploration of individual error patterns suggests that these assessments probe somewhat distinct components of data visualization literacy, and we do not find evidence that these components correspond to the categories that guided the design of either test (e.g., questions that require retrieving values rather than making comparisons). Together, these findings suggest opportunities for development of more comprehensive assessments of data visualization literacy that are organized by components that better account for detailed behavioral patterns.

**Supplementary Information:**

The online version contains supplementary material available at 10.1186/s41235-025-00622-9.

## Significance statement

Data visualizations are indispensable for communicating patterns in quantitative data. However, while several test-based measures of data visualization literacy exist, there is not yet clear agreement on what the key components of data visualization literacy are and how to measure them. In this study, we administered two widely used assessments of data visualization literacy to multiple diverse groups of US adult participants. Participants who performed well on one assessment also generally did so on the other, suggesting some degree of convergence between these two measures. Moreover, performance on the combined assessment was associated with how much formal education in mathematics an individual had received, a measure of their convergence with other measures of quantitative literacy. However, it was less clear what underlying components of data visualization literacy these assessments measure. While it seems natural to assume that the ability to answer any question correctly on this assessment would be predicted by the type of graph shown or the type of question being asked, we used tools from machine learning to discover a small (and different) set of latent factors that could explain these patterns much more effectively. These findings lay the groundwork for future efforts to characterize what aspects of data visualization literacy these latent factors represent and to develop improved and unified measures of data visualization literacy.

## Introduction

Data visualizations—also commonly known as *graphs*, *charts*, and/or *plots*—provide a powerful and versatile medium for reasoning about data (Bertin, 1981; Tufte, 1983; Wilkinson, 2012). They do so by leveraging color, shape, size, position, and other visual variables to convey quantitative patterns and relationships that might otherwise be difficult to discern when inspecting raw data. Although a relatively recent invention (Playfair, 1801; Spence, 2006), data visualizations are now vital for communication both among scientists and between scientists and members of the general public (Börner et al., 2019; Franconeri et al., 2021). As such, the ability to use visualizations to explore and reason about data is a key priority in STEM education (Council, 2014; Garfield and Gal, 1999).

However, a key challenge to successfully addressing this priority is a clear definition of what specific competencies are constitutive of the ability to use data visualizations effectively. This ability, often termed “visualization literacy,” encompasses a broad suite of skills involved in the process of linking questions about data (which are often not inherently visual in nature) to visual patterns in graphical representations of those data (Friel et al., 2001; Shah and Hoeffner, 2002; Brehmer and Munzner, 2013; Boy et al., 2014; Börner et al., 2016; Creamer et al., 2024; Hedayati et al., 2024). Visualization literacy has been operationalized in a number of different ways across disciplinary contexts, including in education (Friel et al., 2001; Shah and Hoeffner, 2002; Maltese et al., 2015; Börner et al., 2019), cognitive psychology (Boy et al., 2014; Padilla, 2018), human–computer interaction (Brehmer and Munzner, 2013; Lee et al., 2016), and public health (Galesic and Garcia-Retamero, 2011; Ancker et al., 2006; Padilla et al., 2022).

While much of the existing work on visualization literacy focuses on the ability to understand formal data visualizations, other lines of work have explored the ability to design new visualizations (Alper et al., 2017; Berg and Smith, 1994; Bishop et al., 2019) or use an existing visualization to make a sound decision (Ruginski et al., 2016; Price et al., 2016). Measures of data visualization understanding are especially important because these comprehension skills are foundational for more complex activities, such as visualization design and decision-making with visualizations (Börner et al., 2019; Hedayati et al., 2024). Moreover, reliable and valid measures of data visualization literacy are critical for evaluating the success of any educational intervention intended to improve visualization literacy skills. Finally, reliable measurement is crucial for developing cognitive theories of data visualization literacy—that is, theories of how graphs are mentally represented that explain why people find some questions about them easier to answer than others, as well as how the ability to understand graphs develops over time (Pinker, 1990; Shah and Hoeffner, 2002; Padilla, 2018; Padilla et al., 2018).

Generally speaking, an individual’s ability to read and interpret a data visualization is assessed using a sequence of test items, each one posing a question and providing a data visualization to answer it. While there are currently several assessments that adopt this general strategy (DelMas et al., 2005; Galesic and Garcia-Retamero, 2011; Maltese et al., 2015; Boy et al., 2014; Lee et al., 2016; Börner et al., 2016; Garcia-Retamero et al., 2016; Okan et al., 2019; Pandey and Ottley, 2023; Ge et al., 2023), they define and operationalize the component skills in different ways. For instance, some assessments group items into a compact hierarchy of abstract abilities, progressing from “reading the data” to “reading beyond the data” (Galesic and Garcia-Retamero, 2011; Friel et al., 2001). Others group items into a broader set of tasks that do not necessarily imply strong dependencies between them, such as finding extreme values or making comparisons (Lee et al., 2016; Pandey and Ottley, 2023; Boy et al., 2014). Still others focus on the ability to overcome intentionally misleading data visualizations (Ge et al., 2023) or misconceptions about distributions that are common among students enrolled in introductory statistics courses (DelMas et al., 2005).

But because these assessments have not been compared directly, it is unknown to what degree they converge with one another or imply the same decomposition of data visualization literacy into underlying skills. As such, it remains unclear on what basis any given assessment should be preferred to provide the most reliable and valid measure of data visualization literacy. To address this gap, here we compare two widely used assessments that measure data visualization literacy in distinct ways: The 13-item assessment developed by Galesic and Garcia-Retamero (2011), which we refer to as *GGR*, and the 53-item Visualization Literacy Assessment Test (*VLAT*; Lee et al. (2016)). We focused on these two assessments because, at the time this work was being conducted, they were among the most influential measures of data visualization literacy that could also be combined into a single assessment that could be administered in one session.

The items in GGR are organized into a three-level hierarchy of skills (Friel et al., 2001): “Level 1: Read the Data” (i.e., finding specific values in a graph); “Level 2: Read Between the Data” (i.e., comparing values in a graph); and “Level 3: Read Beyond the Data” (i.e., extrapolation). On the other hand, the items in VLAT are organized into a suite of eight skills (Brehmer and Munzner, 2013; Amar et al., 2005): retrieving a value, finding extreme values, finding anomalies, making comparisons, determining a range, finding correlations and trends, characterizing distributions, and finding clusters.

We administered both assessments to a large and diverse sample of adult participants in the United States (USA). Our analyses of task performance were guided by three objectives. *First*, to characterize performance on the assessments across different sample demographics, as well as to estimate the association between performance on these assessments and the amount of prior coursework in mathematics. *Second*, we investigated the degree to which these two assessments produced convergent estimates of overall data visualization literacy levels, despite having been designed in different ways. *Third,* we sought to measure how well individual variability in test performance could be explained by the skill-based categories used to design each assessment. Taken together, this study offers empirical insights that serve as a foundation for the future development of more comprehensive and well-validated assessments of data visualization literacy.

## Method

### Participants

A total of 1,176 participants were recruited: 726 were students recruited from the University of California, San Diego study pool (211 male; mean age=21). 450 adults were recruited using Prolific to obtain a sample that is demographically representative of the USA based on age, sex, and ethnicity (206 male; mean age=45). This total sample size is comparable to the study reported in Galesic and Garcia-Retamero (2011), which included 987 participants, and larger than the sample initially recruited in Lee et al. (2016), which included 46 participants. All participants provided informed consent in accordance with the University of California, San Diego IRB. Participants were excluded for failing to complete the full assessment and the post-experiment survey of demographics and prior math experience, as well as for any technical issues reported in the post-experiment survey which prevented them from answering the questions. Unless otherwise indicated, we report results from analyzing the combined sample after exclusions (*N*=1,113 participants; US university sample: *N*=714 participants; US general public: *N*=399 participants).

### Materials

**Fig. 1:**
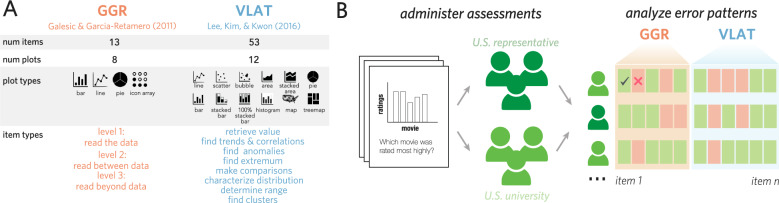
**A** The current study investigates two assessments of data visualization literacy: GGR (Galesic and Garcia-Retamero, 2011) and VLAT (Lee et al., 2016). **B** The combined assessment was administered to two groups of participants: A US university sample recruited using a study pool and a US demographically representative sample recruited using an online crowdsourcing platform

Two assessments were included in our study: *GGR* (Galesic and Garcia-Retamero, 2011) and *VLAT* (Lee et al., 2016); see Fig. 1A.

*GGR* is a 13-item assessment containing eight graphs: three *bar charts*, one *pie chart*, three *line plots*, and one *icon array* (Fig. 1A, left). All items were assigned by the test developers to three categories: *Level 1: Read the Data*, *Level 2: Read Between the Data*, and *Level 3: Read Beyond the Data*. However, not all graph types were paired with all three types of questions. Nine items were fill-in-the-blank questions, and four were multiple-choice questions with three response options. It was possible to skip any multiple-choice question, but not fill-in-the-blank questions, following the original test administration procedure. For all but one of the fill-in-the-blank items, it was necessary to provide a response that exactly matched the correct answer to be counted as correct; for the remaining item, the test developers allowed responses that fell within a range (i.e., between 23 and 25).

*VLAT* is a 53-item assessment containing 12 graph types: *line chart*, *bar chart*, *stacked bar chart*, *100% stacked bar chart*, *pie chart*, *histogram*, *scatter plot*, *bubble chart*, *area chart*, *stacked area chart*, *choropleth map*, and *tree map* (Fig. 1A, right). These items were assigned by the test developers to eight question types: *retrieve value*, *find extremum*, *find anomalies*, *make comparisons*, *determine range*, *find correlations/trends*, *characterize distribution*, and *find clusters*. There were 16 true-false items; the remaining 37 multiple-choice items contained either three options (3 questions) or four options (34 questions). It was possible to skip any question.

### Procedure

Participants completed the two assessments in a randomized order during a single session (see, Fig. 1B), and each assessment was administered in a manner as similar to the original procedure as possible. Participants could spend as much time on each assessment as needed. Afterward, they completed an optional post-study questionnaire that asked about their sex, age, ethnicity, and level of educational attainment. The possible responses to the educational attainment item were: “Have not graduated high school,” “High school graduate, diploma or equivalent,” “Associate degree,” “Bachelor’s degree,” “Master’s degree,” “Professional degree (e.g., M.D., J.D.),” “Doctoral degree (e.g., Ph.D.).” To obtain a proxy for the amount of prior mathematics knowledge participants had, which is especially relevant for tasks involving reasoning about data visualizations, we also prompted participants to indicate how many of the following high school-level math courses they had previously taken: algebra, calculus, and statistics.

**Table 1:** Number of participants grouped by how many high school-level math courses they reported having previously taken

Num math courses	US representative	US university
0	39	9
1	136	46
2	116	146
3	108	513
Total	399	714

### Statistical analyses

Overall, our statistical analyses aim to disentangle different potential sources of variation in how well participants performed on these assessments. The primary tool we use for this is linear regression, and the primary factors we consider are the type of graph used in an item, the type of question asked, prior mathematics knowledge, as well as the population from which participants were recruited (i.e., university study pool vs. demographically representative sample of US crowd workers). To contextualize the degree to which these factors account for the explainable variance in these data, we additionally conducted exploratory factor analysis to infer the latent components that best predict participants’ individual patterns of correct and incorrect responses. Below we describe the details of the statistical analysis strategy we use toward these ends.

#### Linear models

We construct linear models to test the reliability of the association between several different variables of interest (i.e., graph type, question type, number of math courses, group membership) and test performance. We use nested model comparison because it provides a unified framework for hypothesis testing that generalizes beyond the narrower set of use cases that traditional hypothesis tests (e.g., *t*-tests, ANCOVA) were designed to independently handle. Specifically, to estimate the strength of the relationship between each predictor variable of interest and test performance, we fit mixed-effects logistic-regression models to predict accuracy from that predictor variable, modeled as a fixed effect, and include random intercepts for each participant. We use logistic regression to predict the binary outcomes for individual items (i.e., correct vs. incorrect) because it provides more accurate estimates than using ordinary least squares regression to predict the proportion of correct responses across a set of items. To assess the explanatory value of any given predictor variable, we then use nested model comparison to determine how much more variance in performance could be explained when that variable was included in the linear model than when it was omitted (i.e., the baseline model), accounting for the increase in model complexity when the variable was included. We implement these analyses using the *lmer* package in R (Baayen et al., 2008; Bates et al., 2015) and report $\chi ^2$ statistics, degrees of freedom, and p-values for each model comparison. The coefficient and standard error estimates accompanying these models can be found in the Supplemental Materials.

#### Exploratory factor analysis

To investigate latent structure within and between assessments that was predictive of test performance, we employed exploratory factor analysis (EFA). EFA is a widely used dimensionality reduction method to uncover the set of latent *factors* that underlie observable patterns in data (Briggs and Cheek, 1986; Haig, 2005; Cowen and Keltner, 2017; Eisenberg et al., 2019). We apply EFA to the combined assessment in two ways. First, as a tool to infer how many factors are needed to account for the patterns of correct and incorrect responses generated by different participants. Second, we adopt the same formalization to compare existing methods of decomposing assessment items (by question type, graph type, and test) to explain the same error patterns.

To fit a factor model, each response on the combined assessment is modeled as a linear combination of latent factors and measurement error: $X - \mu = LF + \epsilon$, where *X* is an *m* (number of test items: 66) x *n* (number of participants) binary matrix of observed errors and $\mu$ is a matrix containing the mean score for each item. *L* is the *m* x *f* (number of factors) loading matrix, an estimate of how much each item contributes to each latent factor, and *F* is the *f* x *n* matrix of factor scores, an estimate of how much each participant’s responses are predicted by each factor. $\epsilon$ represents measurement error, variance left unexplained by the latent factors.

We first apply EFA to the combined assessment to estimate the number of latent factors needed to account for error patterns while minimizing extraneous factors. Adding more factors improves prediction accuracy, but this often comes at a cost of interpretability. Researchers have proposed several methods for selecting the number of factors that best balances this trade-off in a particular set of data (Preacher et al., 2013). We use Bayesian Information Criterion (BIC) to identify a minimal set of factors that account for individual patterns of error (Schwarz, 1978). We then compare this *latent factor* model to several *idealized* factor models based on graph type, question type, and test.

Rather than estimate the loading matrix *L* from participants’ responses, our idealized factor models specify a loading matrix based on known decompositions of the assessment items. For example, the idealized loading matrix encoding question type information is a 66 x 11 (number of question types) matrix with binary values in each column encoding whether the item in each row belonged to that question type. This specification of *L* embodies the possibility that all of the items that involve the same question type “hang together” to explain error patterns, i.e., an individual who knows how to perform the operation for a particular question type is predicted to get all of those items correct or all of those items incorrect. In addition, encoding each item’s question type independently in the loading matrix *L* allows for the *possibility* that different question types are entirely independent of one another, i.e., an individual who knows how to perform one question type task is not more likely to be able to perform another. Critically, structuring the loading matrix in this way does not *enforce* such independence on the idealized factor models. The idealized factor models are derived by estimating the factor scores *F* given an idealized loading matrix *L*—in this way, systematic patterns in participants’ responses that arise from, e.g., similarity across different question types can be expressed in the factor scores assigned to participants for those question types. Our analyses focus on how well idealized models with “manually” encoded loading matrices and freely varying factor scores are able to predict participants’ responses.

We compare the performance of our fitted latent factor model to idealized factor models encoding test, question type, and graph type information. Each factor model predicts individual responses on all 66 assessment questions. These predictions can be compared to the actual responses to produce a vector of prediction errors for each participant. We calculate each participant’s mean squared error (the average of item-level squared errors for each participant). The average of all participant mean squared error (MSE) values for a given model provides a group-level MSE value for that factor model, allowing us to compare models according to their overall predictive accuracy. For the latent factor model, which does not specify a particular factor loading matrix in advance, we obtain this MSE estimate using fivefold cross-validation. We fit a separate factor model to each set of training data folds, then use the loading matrix from the training set to estimate a factor score matrix for the held-out data. This model is used to then predict individual responses in the held-out data. The group-level MSE value for this model is calculated based on the held-out prediction error for each participant. This ensures that evaluation of model performance is always based on splits of the data that are independent from those used to fit the latent factor model’s loading matrix.

#### Confidence intervals

To provide quantitative estimates of effect size, we report 95% confidence intervals (CIs) for various quantities of interest (e.g., average test performance). Where we have used linear models to fit the data, these confidence intervals were constructed using estimates of standard error based on the linear model itself. Estimates of mean squared error (MSE) for predictions made by our exploratory factor analysis model are calculated with bootstrap resampling methods, which have the advantage of not depending on parametric assumptions about the sampling distribution of the statistic (Efron and Tibshirani, 1994). This approach entailed resampling *N*=1,113 individual participant MSE values with replacement and calculating a group-level MSE value from this sample. We repeated this process 10,000 times to estimate a sampling distribution of group-level MSE values from which the 2.5th and 97.5th percentile values could serve as confidence interval endpoints.

## Results

Our analyses^1^ were guided by three main objectives. First, we sought to estimate differences in performance on the combined assessment between groups of participants, depending on how they were recruited and how much prior coursework in mathematics they had completed, providing initial insights into potential sources of variability in performance across individuals. Second, we assessed how strongly performance on one assessment was associated with performance on the other, providing a preliminary measure of these two assessments’ convergent validity. Third, we evaluated how well variability in performance could be explained by the skill-based categories used to group items in each assessment, such as which type of graph was presented or what type of question was being asked. We compared the predictive value of these skill-based categories to that of an alternative data-driven decomposition, providing a principled way of assessing the reliability of these categories based on their ability to predict individual error patterns.

### Comparing performance across groups

**Fig. 2:**
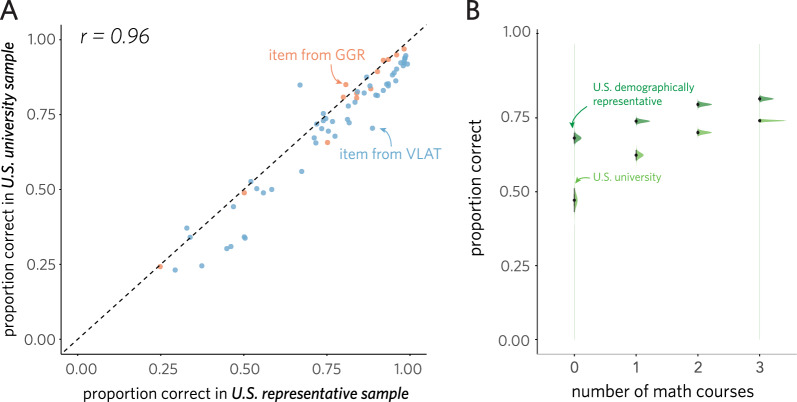
**A** Mean performance on each test item in both US university and US demographically representative samples. Each dot is a test item. **B** Overall performance in each group as a function of the number of math courses (i.e., algebra, calculus, statistics) previously taken. Error bars represent standard error of the mean (SEM)

On average, participants answered 75.7% of test items correctly (95% CI: [74.9%, 76.5%]), though test performance varied substantially across participants (SD: 14.0%; min: 3.0%; max: 97.0%). These findings indicate both that participants were neither at ceiling nor at floor on these assessments, and that there is meaningful individual variability in performance to explain. We also observed that accuracy for the demographically representative sample (78.6%, 95% CI: [77.4%, 79.7%]) was higher on the combined assessment than for the university sample (73.9%, 95% CI: [72.8%, 75.0%]). Nevertheless, we found that relative performance on individual test items was highly correlated between samples ($\rho$ = 0.96, 95% CI = [0.94, 0.98], $p$ < .0001; Fig. 2A). Together, these results suggest that while the two groups of participants performed at different levels on average, data from both samples provide convergent estimates of the test items’ relative difficulty.

We next sought to explore the relationship between performance on these assessments and other relevant characteristics of these participants, with a focus on how much high school-level coursework in mathematics they had completed (Table 1). Toward that end, we grouped participants by how many math courses they reported having previously taken (among algebra, calculus, and statistics). We found that the number of math courses an individual had taken was a reliable predictor of overall test performance ($\chi^2$(3) = 40.04, $p$ < .0001), with more prior math courses leading to higher predicted performance (0: 65.1%, 1: 72.5%, 2: 75.9%, 3: 77.1%); the strength of this association did not differ significantly between sample groups ($\chi^2$(3) = 3.96, $p$ = .27; see Fig. 2B).

**Fig. 3:**
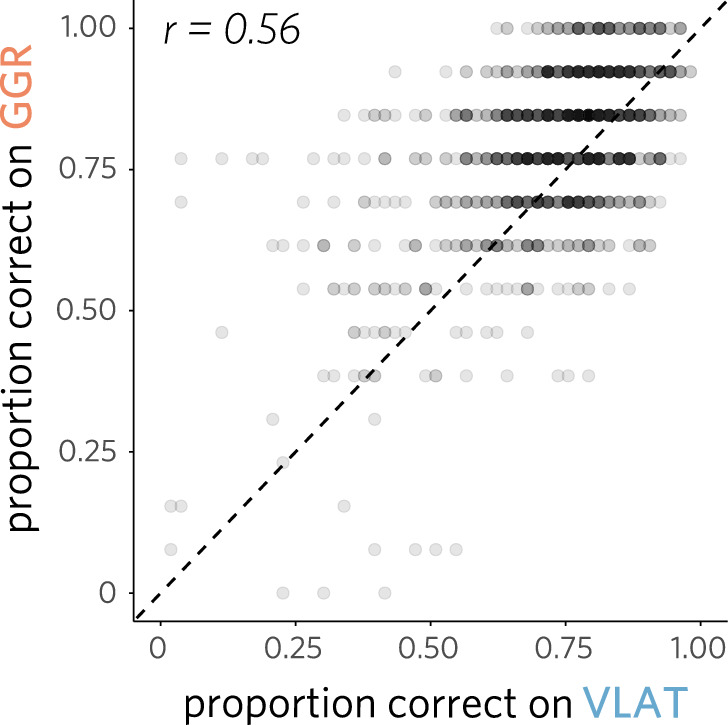
Correlation between performance on VLAT and GGR assessments for individual participants

### Comparing performance across assessments

On average, participants achieved a level of performance that was reliably well above chance, yet below ceiling, on both tests (GGR: 80.4%, 95% CI: [79.5%, 81.3%]; VLAT: 74.6%, 95% CI: [73.7%, 75.5%]). However, there was also substantial individual variability in test performance (GGR *SD* = 15.0%, VLAT *SD* = 15.1%), with an individual’s score on one test being moderately predictive of their score on the other ($\rho$ = 0.56, 95% CI: [0.52, 0.60 ]; Fig. 3). These findings provide an initial estimate of these two assessments’ ability to reliably measure the same construct. Nevertheless, this analysis does not resolve what underlying factors account for the observed level of convergence between assessments and what accounts for the remainder of the gap between them.

**Fig. 4:**
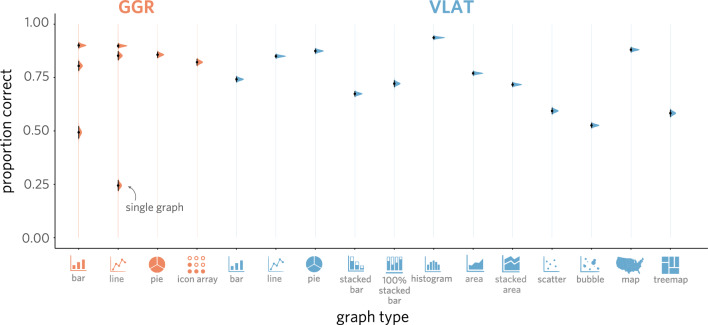
Mean proportion correct for every type of graph in the combined assessment, disaggregated by test. Point estimates are plotted for each individual graph, aggregating questions that pertain to the same graph. GGR contains multiple instances of bar plots and line plots, one pie chart and one icon array. VLAT contains exactly one instance of each type graph. The sampling distributions for each point estimate are shown along with error bars representing the standard error of the mean (SEM)

### Comparing performance across graph type

One possibility that could account for the moderate correlation between scores on each test is the use of similar types of graphs. For instance, some of these graph types might be ones that most individuals know how to interpret, while others are ones that only a minority of individuals are familiar with. Insofar as graph type drives variability in test performance, participants would be expected to achieve higher accuracy on questions involving more familiar graphs, and lower accuracy on questions with less familiar graphs.

**Fig. 5:**
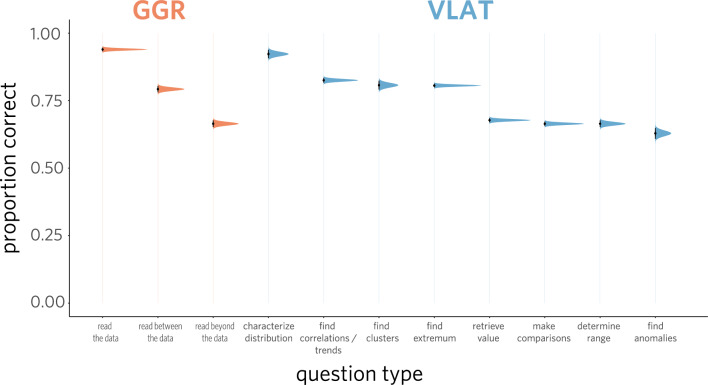
Mean proportion correct for every question type in the combined assessment, disaggregated by test. The sampling distributions for each point estimate are shown along with error bars representing the standard error of the mean (SEM)

To explore that possibility, we took an inventory of the types of graphs appearing in each assessment. We observed that three graph types appeared in both GGR and VLAT (i.e., *bar chart*, *line graph*, and *pie chart*), while there was one additional graph type that appeared only in GGR (i.e., *icon array*) and nine additional graph types appearing only in VLAT (i.e., *stacked bar*, *100% stacked bar*, *histogram*, *area chart*, *stacked area chart*, *scatter plot*, *bubble chart*, *map*, *treemap*). We found that performance reliably varied across graph types in the combined assessment ($\chi^2$(12) = 5066.18, $p$ < .0001; Fig. 4). While both assessments have some overlap in graph types (i.e., bar graphs, line graphs, and pie charts), VLAT uses a broad range of additional graphs; this raises the possibility that the observed effect of graph type on accuracy reflects the combination of the two assessments. However, we find that performance varies significantly by graph type even when considering each assessment individually (*GGR*: $\chi^2$(3) = 125.18, $p$ < .0001; *VLAT*: $\chi^2$(11) = 4919.30, $p$ < .0001). The magnitude of this effect also reliably differed between samples (combined: $\chi^2$(12) = 130.93, $p$ < .0001; GGR: $\chi^2$(3) = 8.67, $p$ = .03; VLAT: $\chi^2$(11) = 112.01, $p$ < .0001), being larger in the demographically representative sample than in the university sample, perhaps reflecting the greater diversity in that sample relative to the university-based sample. Taken together, these results indicate that graph type accounts for a meaningful amount of variation in test performance, suggesting that participants found it easier to answer questions involving some kinds of graphs than others.

### Comparing performance across question type

An additional factor that might account for variation in test performance is the type of question being asked. Perhaps some questions rely on skills that are more broadly shared across participants in the study, such as *Level 1: Read the Data* from GGR and *retrieve value* from VLAT, while other types of questions require understanding of more advanced statistical concepts that are familiar only to a minority of participants, such as the ability to *find correlations/trends* or *characterize distribution* in VLAT.

Insofar as question type is a driver of variability in test performance, participants would be expected to achieve a higher level of accuracy on some types of questions than others. Consistent with this possibility, we found that performance reliably varied by question type, both when each assessment was analyzed independently (*GGR*: $\chi^2$(2) = 1331.61, $p$ < .0001; *VLAT*: $\chi^2$(7) = 1981.92, $p$ < .0001; Fig. 5) and when conducting the same analysis for all 11 question types in both assessments ($\chi^2$(10) = 3585.91, $p$ < .0001). In further exploratory analyses, we found that variation in performance associated with question type was greater in the demographically representative sample (overall: $\chi^2$(10) = 94.18, $p$ < .0001; GGR: $\chi^2$(2) = 1.57, $p$ = .46; VLAT: $\chi^2$(7) = 71.03, $p$ < .0001), perhaps related to the greater heterogeneity in that sample. The finding that question type accounts for variation in test performance suggests that some types of questions are reliably more difficult than others.

### Comparing predictive models of performance

Our findings so far provide evidence that both the type of graph used and the type of question being asked account for at least some of the explainable variance in average performance at the group level. However, an even stronger test of the explanatory value of these factors is their ability to account for variation in the *patterns* of errors that different individuals produce. For instance, insofar as the ability to “read the data” is distinct from the ability to “read beyond the data,” with some individuals having mastered one of these, and other individuals having mastered both, we would expect to be able to predict *which* questions are more likely to be answered correctly by some individuals than others in terms of those skills.

To explore how well graph type and question type predict these individual differences in absolute terms, we sought to establish an upper bound for how well individual error patterns could be explained by any decomposition of these assessments. Toward that end, we used exploratory factor analysis (EFA) to infer the decomposition of the combined assessment that best accounted for observed error patterns across all items (see *Exploratory Factor Analysis* in the Methods section for details).

**Fig. 6:**
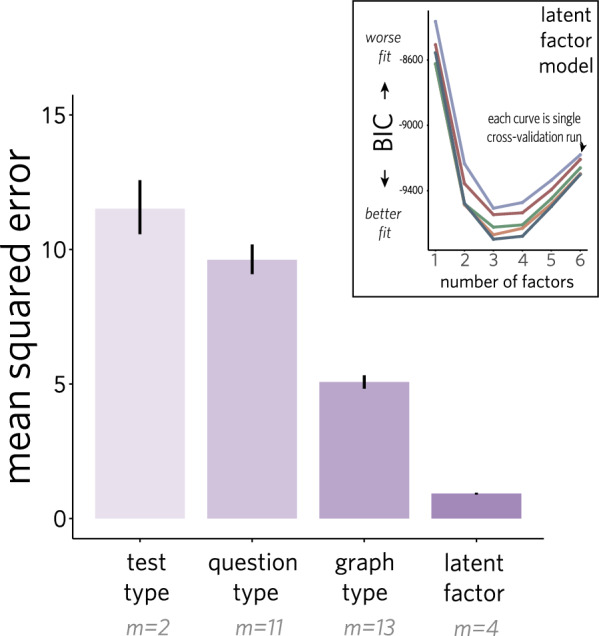
Comparing the ability of different factor-based models to predict individual participant error patterns. Inset indicates number of factors needed by best-performing latent factor model (each curve depicts model fit with increasing factors for a different subset of the data)

First, we investigated how many factors would be needed to achieve strong out-of-sample behavioral predictivity without being too complex. When fitting the data with a variable number of factors, we found that a model with three to four factors consistently achieved the best performance, as measured using Bayesian Information Criterion (BIC; Fig. 6, *inset*). These findings suggest that participants’ error patterns can be explained by a relatively compact set of factors, and far fewer than there are unique types of questions and graphs across VLAT and GGR.

Next, we sought to directly compare the performance of idealized factor models based on graph type (13 factors), question type (11 factors), and test (2 factors) compared with that achieved by this *latent factor* model (4 factors) on held-out data under fivefold cross-validation. We compared the mean squared error (MSE) of the predictions made by each of our models (Fig. 6). We find that both the *question type* (MSE = 9.62, 95% CI: [9.09, 10.19]) and *graph type* (MSE = 5.07, 95% CI: [4.83, 5.32]) models perform better than a 2-factor *test type* model (MSE = 11.52, 95% CI: [10.57, 12.57]), suggesting that these features of the assessment items allow for systematic predictions of participants’ errors relative to the differentiation made by test alone. Further, we find that the 13-factor *graph type* model performs better than the 11-factor *question type* model, suggesting that fluency with some graphs and not others explains more variance in participant responses than their comfort with particular question types (Peebles and Cheng, 2003). Finally, both the *graph type* and *question type* models perform substantially worse than the 4-factor *latent factor* model (MSE = 0.93, 95% CI: [0.89, 0.96]). Taken together, these results suggest that neither graph type nor question type on their own can account for much of the explainable variation in individual error patterns.

**Fig. 7:**
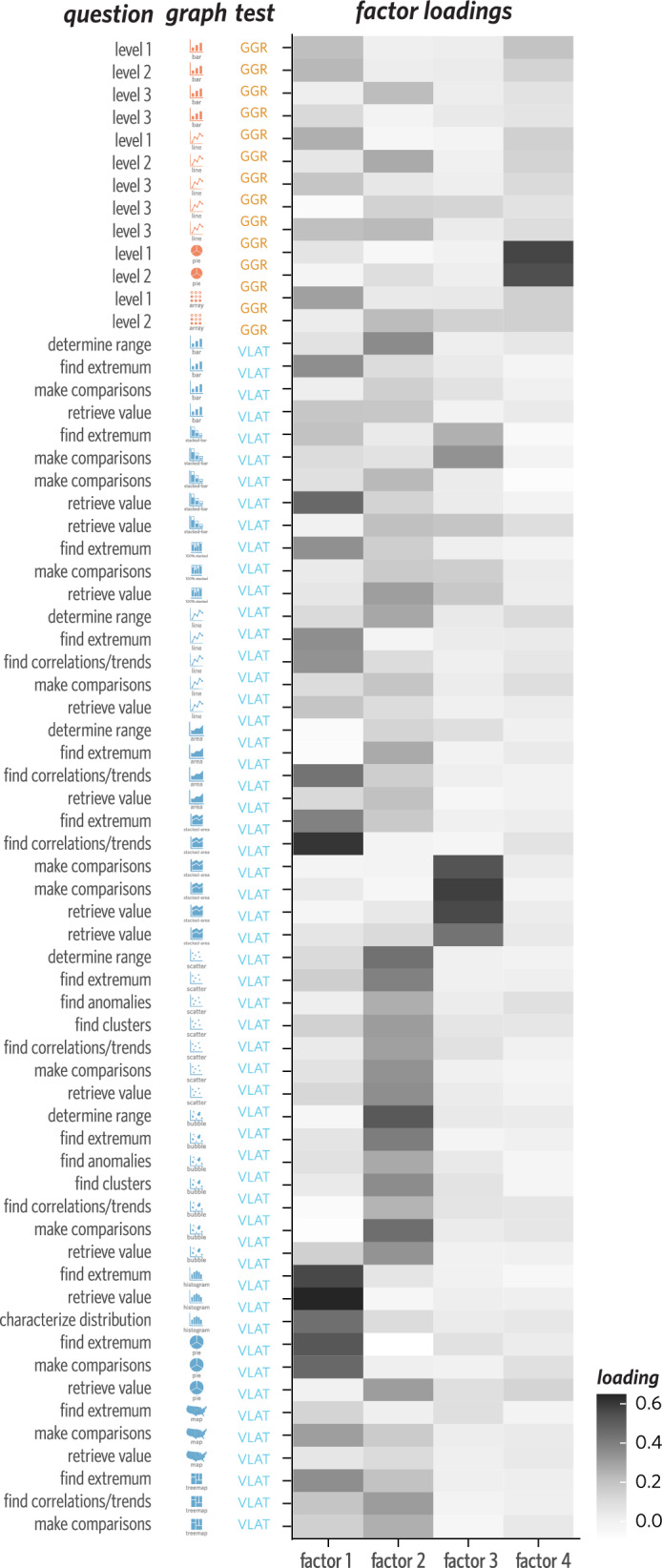
Matrix indicating factor loading values across all items based on the latent factor model, grouped by test, question, and graph type. Darker cells reflect higher factor loading values. See Supplemental Materials for numerical loading values

Instead, these error patterns may reflect a more complex interaction between graph type and question type, which is captured by the *latent factor* model. If that were the case, then some combinations of question type and graph type (e.g., *find extremum* for a *histogram*) would be expected to load strongly on a latent factor, while other items involving either just that question type or just that graph type would not. While items involving scatterplots and bubble charts seem to load onto factor 2, perhaps reflecting the similarity between these types of graphs, it is far less clear what unifies the items that load onto the remaining factors (Fig. 7; see Supplemental Materials for numerical loading values). Indeed, it seems possible that there are features of these items beyond graph type and question type information that might be needed to better explain these behavioral data. In sum, a small number of factors seems sufficient to account for individual error patterns across VLAT and GGR, but these factors do not obviously reflect the categories often used to differentiate items in these assessments.

## Discussion

In this study, we administered two widely used assessments of data visualization literacy (Galesic and Garcia-Retamero, 2011; Lee et al., 2016) to multiple independently recruited samples of US adult participants. Participants who performed well on one assessment also generally performed well on the other, suggesting some degree of convergence between these two measures. Moreover, performance on the combined assessment was associated with how much formal education in mathematics participants had received, an indicator of these assessments’ convergence with other measures of quantitative literacy. However, further investigation of individual variability in the patterns of mistakes that participants made suggests that these assessments probe a suite of skills that are only partially aligned with the grouping of items according to the type of question being asked or the type of graph being shown. That is, while there is some variance explained by these two variables, there remains substantial variance that remains unexplained by them and is better explained by an alternative set of factors that does not seem to have been explicitly encoded into the design of these two assessments.

To make sense of these results, it could be helpful to draw a distinction between an abstract framework for organizing the space of skills relevant to data visualization literacy (i.e., graph type, question type) and the concrete measures used to probe that space of skills. Our results could imply limitations in the measures, the underlying framework, or both. However, it is often not feasible to distinguish between these possibilities on the basis of any individual study, including the current one.

Nevertheless, clarifying these issues will likely involve conducting more thorough evaluations that include an expanded set of measures. For example, it might be useful to include assessments that focus on the ability to overcome potentially misleading data visualizations (Ge et al., 2023), as well as specific misconceptions that are common among students enrolled in introductory statistics courses (DelMas et al., 2005). However, even this broader set of existing assessments is still limited in several key ways. First, there are only a few examples of each type of graph represented across all of them. As such, the type of graph is almost always confounded with the variables being plotted, leaving it unclear whether variation in performance is attributable to core visualization literacy skills, rather than other factors (e.g., prior knowledge about those variables). Second, each test contains a relatively small and fixed set of items. With so few items, it is challenging to estimate the reliability with which any given skill is being measured. With only fixed sets of items, it is also not feasible to measure changes in data visualization literacy within the same individual, which is critical for assessing the impact of formal instruction.

Thus, it would be valuable to develop new assessments, leveraging both best practices in psychometric research already exemplified by the design of existing tests (Boy et al., 2014; Lee et al., 2016), as well as practical strategies from modern machine learning for scaling the generation of test items for inclusion in cognitive assessments (Methani et al., 2020; Masry et al., 2022; Zelikman et al., 2023). In addition, given the broad set of skills that are recruited when interpreting a data visualization, it can be cumbersome to administer complete versions of every test, if the goal is to quickly assess an individual’s literacy level. A more efficient alternative might be to use a more targeted set of items identified using item-response theory that are particularly diagnostic (Pandey and Ottley, 2023), or even use adaptive testing protocols that dynamically propose sequences of items to administer that will be most informative about an individual’s literacy level given their responses so far (Cui et al., 2023). The approach taken in the current study could be used in conjunction with these more targeted and adaptive test development strategies to identify multiple facets of visualization literacy that would be valuable to estimate, which would entail going beyond conceptualizing literacy level as varying along a single dimension. Such improved assessments would be valuable for advancing educational assessment—the understanding of how well core data literacy skills are being learned in real-world educational settings.

Separately, our findings are also consistent with the possibility that there might be alternative frameworks for decomposing data visualization literacy that provide both more detailed and generalizable ways of predicting quantitative patterns in task performance. For instance, recent work employing qualitative methods to analyze process-level barriers to correct interpretation of data visualizations in VLAT has emphasized distinctions between errors in translating verbal questions into visual queries and errors in the interpretation of plot elements (Nobre et al., 2024). Extending such process-level analyses might be a promising route toward clarifying the relationship between the empirically derived decomposition uncovered in the current study and the typologies of data visualization literacy skills proposed in prior work (Friel et al., 2001; Brehmer and Munzner, 2013; Börner et al., 2019). Measuring those component skills is important because they enable differentiation between individuals who might otherwise seem equally proficient, but actually have different strengths and weaknesses. Reliably diagnosing those strengths and weaknesses makes it possible to then provide instruction that is more effectively tailored to each individual.

Beyond their role in educational assessment and instruction, new measures of data visualization literacy could also be instrumental for advancing fundamental understanding of the cognitive processes involved in the successful interpretation of data visualizations (Pinker, 1990; Padilla, 2018; Shah and Hoeffner, 2002). These processes include the rapid perceptual computations (Cleveland and McGill, 1984) performed with respect to a known graph schema (Pinker, 1990), explicit numerical operations (Gillan and Lewis, 1994) constrained by fintite working memory resources (Padilla et al., 2018), and interpretive processes that lead to more general insights (Carpenter and Shah, 1998), which may be influenced by prior content knowledge (Shah and Freedman, 2011). A more thorough understanding of each of these cognitive processes is a crucial step toward more unified cognitive models of data visualization understanding. One important purpose of such cognitive models is to explain why someone finds some questions easier to answer with one graph than another (Shah and Hoeffner, 2002; Huey et al., 2023). Previously developed cognitive models have proposed qualitative accounts of how people reason about data visualizations (Carpenter and Shah, 1998; Padilla et al., 2018). A promising avenue for future work is to develop *computational* cognitive models that specify the operations performed in explicit and quantitative terms: the form of the input, the form of the output, and the exact operations applied in between. Computational cognitive models have enabled major advances across several cognitive domains because they not only offer precise specifications of the mental processes involved, but also generate concrete behavioral outputs that can be directly compared to what people produce given the same inputs (Peterson et al., 2021; Cao and Yamins, 2024; Bear et al., 2021; Hu et al., 2022; Mukherjee et al., 2024). Future work employing these approaches are especially timely, as computational cognitive models—and in particular, AI systems that perform complex real-world tasks—have only recently advanced to the point that it is feasible to measure these models’ behavior on tasks that approach the complexity of those that humans encounter in real-world settings (Bommasani et al., 2021).

In the current study, we measured a positive relationship between the number of mathematics courses an individual had previously taken and how well they performed on the combined assessment, consistent with prior work examining the association between formal education and behavior on tasks involving data visualizations (Maltese et al., 2015; Harsh et al., 2019). However, while such correlative findings are suggestive, experimental studies are needed to firmly establish any causal relationships between specific learning experiences and subsequent task performance (Koedinger et al., 2023; Bhatt et al., 2024; Solomon et al., 2019). A promising approach complementing studies with real students might be to conduct so-called *in silico* experiments with computational cognitive models. These models can be used to develop and test hypotheses about what kinds of experience are needed to acquire various data visualization literacy skills. A major advantage of *in silico* experiments is that they enable researchers to efficiently sweep through a wider range of possible learning conditions than can be practically and ethically implemented in real-world educational environments with human learners. By systematically manipulating the amount and type of experience a computational model receives, it is possible to investigate what kinds of experience are needed to succeed on some tasks and generalize to others (Zamir et al., 2018; Zhuang et al., 2021; Gupta et al., 2024). For example, a vision-language model might be pretrained on a suite of visual, language-based, and quantitative reasoning tasks before being evaluated on its ability to accurately interpret data visualizations (Gupta et al., 2024). Comparisons between this model and others that had been pretrained on only a subset of the same tasks could be used to assess the necessity of certain kinds of prior experience to generalize to reasoning tasks involving data visualizations.

## Conclusions

In sum, the current study evaluated the convergent validity of two commonly used assessments of data visualization literacy. We found that these two measures exhibited a reasonable degree of convergence, such that people who achieved a high score on one assessment often did so on the other as well. In addition, we observed that individual variability in performance was related to how much formal education in mathematics an individual had received. To gain insight into the component skills that underlie the observed relationship between assessments, we used tools from machine learning to identify latent factors that best predicted individual error patterns. These latent factors achieve high predictive accuracy but do not align with existing typologies that have been used to structure these assessments (i.e., the type of graph, the kind of task being performed), suggesting the need for future work to characterize what component skills these factors identify. In sum, this work lays the groundwork for future efforts to develop improved and unified assessments that provide accurate and reliable estimates of the underlying components of data visualization literacy (Uttal et al., 2024). Over the long run, the development of unified measures of data visualization literacy is not only crucial for advancing cognitive theories of how people learn to extract meaning from abstract graphical representations, but might also lead to improved ways of teaching graphical literacy skills in real-world educational settings.

## Open Practices

All data reported in this manuscript, along with analysis scripts used to generate the current results, are publicly available at the following GitHub repository: https://github.com/cogtoolslab/visualization_literacy_convergent_validity.

## Supplementary Information

Supplementary file 1.

## Data Availability

All data described in this manuscript, along with analysis scripts used to generate the current results, are publicly available at the following GitHub repository: https://github.com/cogtoolslab/visualization_literacy_convergent_validity.

## Footnotes

1 All data, along with analysis scripts used to generate the current results, are publicly available at the following GitHub repository: https://github.com/cogtoolslab/visualization_literacy_convergent_validity.
