# Supplementary material for: Evaluating convergence between two data visualization literacy assessments
Source: Cogn Res Princ Implic. 2025 Apr 5;10:15. doi: 10.1186/s41235-025-00622-9 (PMC11972256; doi:10.1186/s41235-025-00622-9)
Supplement: Supplementary file 1 [file 41235_2025_622_MOESM1_ESM.pdf]

SUPPLEMENT for Evaluating convergence between two data visualization literacy assessments

1 Model fits

Table 1 Logistic model predicting `accuracy (0/1)` ∼ `prior math` (values below are in logit space)

Math experience	Estimate	SE
0 (intercept)	0.6249	0.1039
1	0.3462	0.1168
2	0.5222	0.1134
3	0.5913	0.1080

Table 2 Logistic model predicting `accuracy (0/1)` ∼ `prior math * sample` (values below are in logit space)

Math experience * Sample	Estimate	SE
0 math (intercept)	0.8084	0.1121
1 math	0.3055	0.1271
2 math	0.6312	0.1301
3 math	0.7838	0.1320
university	-0.9689	0.2617
1 math * university	0.4061	0.2870
2 math * university	0.4474	0.2761
3 math * university	0.5130	0.2729

Table 3 Logistic model predicting `accuracy (0/1)` ∼ `graph type` (values below are in logit space)

Graph type	Estimate	SE
area (intercept)	0.41085	0.03904
bar chart	0.85205	0.04131
bubble chart	0.61500	0.04159
choropleth	-0.04981	0.04898
histogram	2.54428	0.07995
icon array	1.27909	0.06615
line chart	1.15070	0.04075
normalized stacked bar chart	0.64002	0.05175
pie chart	1.66087	0.05223
scatterplot	0.92403	0.04277
stacked area chart	-0.30826	0.04121
stacked bar chart	0.38716	0.04398
treemap	1.78867	0.06399

Table 4 Logistic model predicting `accuracy (0/1)` ∼ `graph type` for **GGR only** (values below are in logit space)

Graph type	Estimate	SE
bar chart (intercept)	1.32918	0.04168
icon array	0.31166	0.06742
line chart	-0.09688	0.04885
pie chart	0.58603	0.07168

Table 5 Logistic model predicting `accuracy (0/1)` ∼ `graph type` for **VLAT only** (values below are in logit space)

Graph type	Estimate	SE
area (intercept)	0.41399	0.04039
bar chart	0.76605	0.04861
bubble chart	0.62463	0.04192
choropleth	-0.05057	0.04934
histogram	2.58524	0.08071
line chart	1.52779	0.05117
normalized stacked bar chart	0.65004	0.05215
pie chart	1.75577	0.06370
scatterplot	0.93858	0.04315
stacked area chart	-0.31297	0.04154
stacked bar chart	0.39320	0.04435
treemap	1.81728	0.06462

Table 6 Logistic model predicting `accuracy (0/1)` ∼ `graph type * sample` (values below are in logit space)

Graph type	Estimate	SE
area (intercept)	0.56540	0.06451
bar chart	0.86980	0.06957
bubble chart	0.71080	0.07033
choropleth	-0.09702	0.08134
histogram	3.49009	0.20421
icon array	1.36934	0.11581
line chart	1.00728	0.06756
normalized stacked bar chart	0.77541	0.08878
pie chart	1.68978	0.09025
scatterplot	0.79614	0.07086
stacked area chart	-0.15248	0.06862
stacked bar chart	0.41748	0.07382
treemap	2.34566	0.13168
university	-0.24106	0.08064
bar chart * university	-0.02767	0.08634
bubble chart * university	-0.14496	0.08710
choropleth * university	0.07344	0.10167
histogram * university	-1.20669	0.22286
icon array * university	-0.13426	0.14097
line chart * university	0.21797	0.08452
normalized stacked bar chart * university	-0.20359	0.10918
pie chart * university	-0.04424	0.11057
scatterplot * university	0.19502	0.08862
stacked area chart * university	-0.24198	0.08569
stacked bar chart * university	-0.04670	0.09179
treemap * university	-0.75877	0.15133

Table 7 Logistic model predicting `accuracy (0/1)` ∼ `graph type * sample` for **GGR only** (values below are in logit space)

Graph type	Estimate	SE
bar chart (intercept)	1.36140	0.06928
icon array	0.53750	0.11940
line chart	-0.13337	0.08183
pie chart	0.67028	0.12354
university	-0.05058	0.08573
icon array * university	-0.33698	0.14483
line chart * university	0.05681	0.10199
pie chart * university	-0.12810	0.15171

Table 8 Logistic model predicting `accuracy (0/1)` ∼ `graph type * sample` for **VLAT only** (values below are in logit space)

Graph type	Estimate	SE
area (intercept)	0.57281	0.06686
bar chart	0.92974	0.08393
bubble chart	0.71768	0.07102
choropleth	-0.09803	0.08212
histogram	3.51048	0.20553
line chart	1.41412	0.08566
normalized stacked bar chart	0.78281	0.08973
pie chart	1.84189	0.11297
scatterplot	0.80373	0.07165
stacked area chart	-0.15412	0.06935
stacked bar chart	0.42173	0.07448
treemap	2.36208	0.13213
university	-0.24785	0.08341
bar chart * university	-0.24617	0.10288
bubble chart * university	-0.14224	0.08786
choropleth * university	0.07406	0.10255
histogram * university	-1.18538	0.22397
line chart * university	0.16718	0.10656
normalized stacked bar chart * university	-0.20129	0.11023
pie chart * university	-0.13015	0.13662
scatterplot * university	0.20464	0.08953
stacked area chart * university	-0.24661	0.08650
stacked bar chart * university	-0.04472	0.09254
treemap * university	-0.74686	0.15198

Table 9 Logistic model predicting `accuracy (0/1)` ∼ `question type` (values below are in logit space)

Graph type	Estimate	SE
characterize distribution (intercept)	2.7075	0.1195
determine range	-1.9606	0.1212
find anomalies	-2.1339	0.1263
find clusters	-1.1328	0.1300
find correlations / trends	-1.0003	0.1221
find extremum	-1.1422	0.1196
read the data (level 1)	0.2802	0.1341
read between the data (level 2)	-1.2330	0.1237
read beyond the data (level 3)	-1.9606	0.1212
make comparisons	-1.9610	0.1190
retrieve value	-1.8948	0.1190

Table 10 Logistic model predicting `accuracy (0/1)` ∼ `question type` for **GGR only** (values below are in logit space)

Graph type	Estimate	SE
read the data (level 1) (intercept)	2.95645	0.06894
read between the data (level 2)	-1.48425	0.07438
read beyond the data (level 3)	-2.20358	0.07112

Table 11 Logistic model predicting `accuracy (0/1)` ∼ `question type` for **VLAT only** (values below are in logit space)

Graph type	Estimate	SE
characterize distribution (intercept)	2.7272	0.1142
determine range	-1.9750	0.1158
find anomalies	-2.1496	0.1208
find clusters	-1.1410	0.1250
find correlations / trends	-1.0075	0.1168
find extremum	-1.1505	0.1141
make comparisons	-1.9755	0.1135
retrieve value	-1.9087	0.1134

Table 12 Logistic model predicting `accuracy (0/1)` ∼ `question type * sample` (values below are in logit space)

Graph type	Estimate	SE
characterize distribution (intercept)	3.38908	0.22127
determine range	-2.50579	0.22401
find anomalies	-2.80788	0.23020
find clusters	-1.10865	0.24574
find correlations / trends	-1.63136	0.22552
find extremum	-1.53464	0.22195
read the data (level 1)	-0.34593	0.24497
read between the data (level 2)	-1.81822	0.22778
read beyond the data (level 3)	-2.61646	0.22304
make comparisons	-2.37752	0.22058
retrieve value	-2.41337	0.22041
university	-0.95000	0.25621
determine range * university	0.73673	0.25981
find anomalies * university	0.93762	0.26884
find clusters * university	-0.06137	0.28393
find correlations / trends * university	0.86528	0.26179
find extremum * university	0.51017	0.25682
read the data (level 1) * university	0.85220	0.28691
read between the data (level 2) * university	0.79603	0.26500
read beyond the data (level 3) * university	0.90842	0.25897
make comparisons * university	0.54096	0.25526
retrieve value * university	0.69587	0.25506

Table 13 Logistic model predicting `accuracy (0/1)` ∼ `question type * sample` for **GGR only** (values below are in logit space)

Graph type	Estimate	SE
read the data (level 1) (intercept)	3.04049	0.11687
read between the data (level 2)	-1.45966	0.12811
read beyond the data (level 3)	-2.26019	0.12139
university	-0.12996	0.14232
read between the data (level 2) * university	-0.03797	0.15705
read beyond the data (level 3) * university	0.08685	0.14873

Table 14 Logistic model predicting `accuracy (0/1)` ∼ `question type * sample` for **VLAT only** (values below are in logit space)

Graph type	Estimate	SE
characterize distribution (intercept)	3.3977	0.2160
determine range	-2.5084	0.2185
find anomalies	-2.8118	0.2256
find clusters	-1.1084	0.2405
find correlations / trends	-1.6315	0.2199
find extremum	-1.5347	0.2164
make comparisons	-2.3796	0.2154
retrieve value	-2.4156	0.2149
university	-0.9398	0.2489
determine range * university	0.7244	0.2524
find anomalies * university	0.9259	0.2623
find clusters * university	-0.0714	0.2765
find correlations / trends * university	0.8590	0.2542
find extremum * university	0.5016	0.2493
make comparisons * university	0.5276	0.2481
retrieve value * university	0.6836	0.2476

	factor1	factor2	factor3	factor4
GGR, bar chart, level 1, item 1	0.25	0	0.01	0.23
GGR, bar chart, level 2, item 2	0.28	0.01	0.02	0.15
GGR, bar chart, level 3, item 10	0	0.26	0.01	0.07
GGR, bar chart, level 3, item 13	0.12	-0.03	0.03	0.06
GGR, line chart, level 1, item 5	0.3	-0.06	-0.04	0.16
GGR, line chart, level 2, item 6	0.05	0.32	-0.01	0.15
GGR, line chart, level 3, item 7	0.22	0.04	0.01	0.12
GGR, line chart, level 3, item 11	-0.1	0.16	0.14	0.07
GGR, line chart, level 3, item 12	0.24	0.27	0.02	0.1
GGR, pie chart, level 1, item 3	0.06	-0.07	-0.02	0.69
GGR, pie chart, level 2, item 4	-0.05	0.1	0.01	0.66
GGR, icon array, level 1, item 8	0.35	0.03	0.04	0.16
GGR, icon array, level 2, item 9	0.01	0.26	0.16	0.17
VLAT, bar chart, determine range, item 21	0.07	0.43	0.01	0.04
VLAT, bar chart, find extremum, item 20	0.42	0.1	0.02	-0.04
VLAT, bar chart, make comparisons, item 22	0.01	0.17	0.07	-0.01
VLAT, bar chart, retrieve value, item 19	0.21	0.21	-0.03	0.02
VLAT, stacked bar chart, find extremum, item 25	0.23	0.02	0.3	-0.09
VLAT, stacked bar chart, make comparisons, item 27	0.11	0.07	0.4	-0.04
VLAT, stacked bar chart, make comparisons, item 28	0.09	0.27	0.02	-0.11
VLAT, stacked bar chart, retrieve value, item 23	0.56	0.15	0.02	-0.03
VLAT, stacked bar chart, retrieve value, item 24	-0.01	0.24	0.22	0.1
VLAT, normalized stacked bar chart, find extremum, item 30	0.41	0.17	0	-0.03
VLAT, normalized stacked bar chart, make comparisons, item 31	0.02	0.2	0.17	0.01
VLAT, normalized stacked bar chart, retrieve value, item 29	0.05	0.36	0.2	0.01
VLAT, line chart, determine range, item 16	0.12	0.33	0.03	0.11
VLAT, line chart, find extremum, item 15	0.42	-0.04	0.02	0.03
VLAT, line chart, find correlations trends, item 17	0.4	0.11	0	0.05
VLAT, line chart, make comparisons, item 18	0.1	0.22	0.01	0.09
VLAT, line chart, retrieve value, item 14	0.22	0.11	-0.01	0.02
VLAT, area chart, determine range, item 49	-0.1	0.15	0.09	-0.01
VLAT, area chart, find extremum, item 48	-0.1	0.32	-0.02	0.03
VLAT, area chart, find correlations trends, item 50	0.52	0.18	-0.01	-0.03
VLAT, area chart, retrieve value, item 47	0.13	0.24	-0.07	-0.02
VLAT, stacked area chart, find extremum, item 54	0.47	0.2	-0.01	0.01
VLAT, stacked area chart, find correlations trends, item 56	0.74	-0.03	-0.05	0.07
VLAT, stacked area chart, make comparisons, item 57	-0.05	-0.04	0.64	0.01
VLAT, stacked area chart, make comparisons, item 58	0.03	-0.07	0.71	-0.04
VLAT, stacked area chart, retrieve value, item 52	-0.05	0.04	0.68	0.01
VLAT, stacked area chart, retrieve value, item 53	0.05	0.11	0.52	0.03
VLAT, scatterplot, determine range, item 41	0.12	0.53	-0.01	-0.03
VLAT, scatterplot, find extremum, item 40	0.18	0.47	-0.01	0
VLAT, scatterplot, find anomolies, item 43	0	0.31	0.01	0.08
VLAT, scatterplot, find clusters, item 44	0.16	0.37	0.05	0.05
VLAT, scatterplot, find correlations trends, item 45	0.03	0.35	0.07	-0.01
VLAT, scatterplot, make comparisons, item 46	0.07	0.4	-0.01	-0.04
VLAT, scatterplot, retrieve value, item 39	0.13	0.42	0.02	-0.02
VLAT, bubble chart, determine range, item 61	-0.06	0.63	0.03	0.02
VLAT, bubble chart, find extremum, item 60	0.06	0.48	-0.04	-0.01
VLAT, bubble chart, find anomolies, item 63	0.08	0.33	0.03	-0.05
VLAT, bubble chart, find clusters, item 64	0.03	0.42	0.07	-0.05
VLAT, bubble chart, find correlations trends, item 65	-0.09	0.29	0.06	0.05
VLAT, bubble chart, make comparisons, item 66	-0.12	0.54	0.02	0.05
VLAT, bubble chart, retrieve value, item 59	0.17	0.4	-0.02	-0.01
VLAT, histogram, find extremum, item 36	0.68	0.05	-0.01	-0.08
VLAT, histogram, retrieve value, item 35	0.78	-0.06	0.01	-0.01
VLAT, histogram, characterize distribution, item 38	0.53	0.1	0.02	0.04
VLAT, pie chart, find extremum, item 33	0.63	-0.14	0.08	0.02
VLAT, pie chart, make comparisons, item 34	0.56	0	-0.02	0.09
VLAT, pie chart, retrieve value, item 32	-0.02	0.36	0.09	0.15
VLAT, choropleth, find extremum, item 68	0.15	0	0.09	-0.03
VLAT, choropleth, make comparisons, item 69	0.36	0.19	0.01	0.03
VLAT, choropleth, retrieve value, item 67	0.05	0.11	0	0.03
VLAT, treemap, find extremum, item 71	0.42	0.23	0	0.01
VLAT, treemap, find correlations trends, item 73	0.22	0.37	-0.03	0
VLAT, treemap, make comparisons, item 72	0.16	0.3	-0.07	0.04
